# Alzheimer's disease is associated with changes in the metabotropic glutamate mGlu3 receptor expression but not with SNPs in the GRM3 gene

**DOI:** 10.1002/alz70856_103132

**Published:** 2025-12-25

**Authors:** Eugenia Olivera, Pilar Garaventa, Albany Saez, Ana Clara Romero, Daniela Zelaya, Mercedes Bigi, Carla Caruso, Juan Beauquis, Flavia Saravia, Mercedes Lasaga, Daniela Durand

**Affiliations:** ^1^ INBIOMED CONICET, Ciudad Autonoma de Buenos Aires, CABA, Argentina; ^2^ INBIOMED CONICET, Buenos Aires, CABA, Argentina; ^3^ University of Buenos Aires, Buenos Aires, Argentina; ^4^ CONICET, Buenos Aires, Argentina

## Abstract

**Background:**

Our group has demonstrated that the subtype 3 metabotropic glutamate receptor (mGlu3R) expressed in astrocytes exerts neuroprotective functions and promotes both non‐amyloidogenic cleavage of APP and Aβ clearance. In turn, we showed that mGlu3R levels progressively decreased with age in the hippocampus of PDAPP‐J20 murine AD model. The goal of this study was to investigate whether these changes reflect glial dysfunction and are also present in AD patients.

**Method:**

Brain hemispheres‐ derived glial cells from young adult (2 months‐old) PDAPP‐J20 mice or non‐transgenic littermates were cultured after isolation in a Percoll gradient. After 8‐10 days in vitro, cells were lysed to obtain proteins for determination of mGlu3R, GLT‐1, and SR‐A levels for western blot. We also performed bioinformatics analysis of 6 RNASeq databases from brain tissue from patients with AD or controls, using the NCBI online tool GEO2R, in order to study mRNA expression of mGlu3R and other proteins involved in mGlu3R pathway, such as GLT‐1, SRA‐1, and BDNF. TPM (transcripts per million kilobase) data were used to perform logistic regression and ROC curves. For the SNPs analysis, we used RStudio to perform regression and clustering models.

**Result:**

In accordance with reduced hippocampal expression of mGlu3R in PDAPP‐J20 mice, glial mGlu3R levels were early diminished in these animals. This was accompanied with reduced levels of GLT‐1 and increased expression of SR‐A. By analyzing RNASeq databases from AD or control brains, we observed a significant decrease in mGlu3R, GLT1, and BDNF levels in AD patients; while SRA was increased. ROC curve analysis yielded significant results for both mGlu3R and the 4‐gene panel as predictors for AD. Moreover, mGlu3R reduced mRNA levels were mainly linked to the peri‐plaque regions. However, neither GRM3 nor FOLH1 SNPs were correlated to AD diagnostic or to cognitive impairment, after analysis of GWAS data from an Argentinian cohort.

**Conclusion:**

We suggest that early changes in glial mGlu3R expression in AD deprive the brain of the neuroprotective and anti‐amyloidogenic mechanisms executed by the receptor, and this may be involved in the etiology of the disease.

